# The Importance of Visit Notes on Patient Portals for Engaging Less Educated or Nonwhite Patients: Survey Study

**DOI:** 10.2196/jmir.9196

**Published:** 2018-05-24

**Authors:** Macda Gerard, Hannah Chimowitz, Alan Fossa, Fabienne Bourgeois, Leonor Fernandez, Sigall K Bell

**Affiliations:** ^1^ Wayne State University School of Medicine Detroit, MI United States; ^2^ Department of Medicine Beth Israel Deaconess Medical Center Boston, MA United States; ^3^ Department of Medicine Boston Children's Hospital Harvard Medical School Boston, MA United States

**Keywords:** patient engagement, vulnerable populations, patient portals, electronic health record

## Abstract

**Background:**

OpenNotes, a national initiative to share clinicians’ visit notes with patients, can improve patient engagement, but effects on vulnerable populations are not known very well.

**Objective:**

Our aim is to examine the importance of visit notes to nonwhite and less educated patients.

**Methods:**

Patients at an urban academic medical center with an active patient portal account and ≥1 available ambulatory visit note over the prior year were surveyed during June 2016 until September 2016. The survey was designed with patients and families and assessed importance of reading notes (scale 0-10) for (1) understanding health conditions, (2) feeling informed about care, (3) understanding the provider’s thought process, (4) remembering the plan of care, and (5) making decisions about care. We compared the proportion of patients reporting 9-10 (extremely important) for each item stratified by education level, race/ethnicity, and self-reported health. Principal component analysis and correlation measures supported a summary score for the 5 items (Cronbach alpha=.93). We examined factors associated with rating notes as extremely important to engage in care using logistic regression.

**Results:**

Of 24,722 patients, 6913 (27.96%) completed the survey. The majority (6736/6913, 97.44%) read at least one note. Among note readers, 74.0% (727/982) of patients with ≤high school education, 70.7% (130/184) of black patients, and 69.9% (153/219) of Hispanic/Latino patients reported that notes are extremely important to feel informed about their care. The majority of less educated and nonwhite patients reported notes as extremely important to remember the care plan (62.4%, 613/982 ≤high school education; 62.0%, 114/184 black patients; and 61.6%, 135/219 Hispanic/Latino patients) and to make care decisions (62.3%, 612/982; 59.8%, 110/184; and 58.5%, 128/219, respectively, and *P*<.003 for all comparisons to more educated and white patients, respectively). Among patients with the poorest self-reported health, 65.9% (499/757) found notes extremely important to be informed and to understand the provider. On multivariable modeling, less educated patients were nearly three times as likely to report notes were extremely important to engage in care compared with the most educated patients (odds ratio [OR] 2.9, 95% CI 2.4-3.3). Nonwhite patients were twice as likely to report the same compared with white patients (OR 2.0, 95% CI 1.5-2.7 [black] and OR 2.2, 95% CI 1.6-2.9 [Hispanic/Latino and Asian], *P*<.001 for each comparison). Healthier patients, women, older patients, and those who read more notes were more likely to find notes extremely important to engage in care.

**Conclusions:**

Less educated and nonwhite patients using the portal each assigned higher importance to reading notes for several health behaviors than highly educated and white patients, and may find transparent notes especially valuable for understanding their health and engaging in their care. Facilitating access to notes may improve engagement in health care for some vulnerable populations who have historically been more challenging to reach.

## Introduction

As patients seek access to their health information, electronic health records (EHRs) have become increasingly central to health care delivery [1]. Centers for Medicare and Medicaid Services meaningful use incentives have prompted a rapid rise in the number of health care organizations that have implemented EHRs and accompanying Web-based patient portals to increase patient engagement [2]. Among patients engaging with their electronic health data, portal use has been associated with clinical benefits, such as increased rates of diabetes-related medication adherence [3,4]. In addition, patients who access visit notes on the portal report better understanding of their health conditions, feeling more prepared for visits, feeling more in control of health care, better remembering the care plan, and better taking medications as prescribed [5]. Similar results have been reported among US veterans, a population that may represent older, low income, or chronically ill Americans [6,7]. However, potential benefits derived from portal use may be influenced by many factors, including education, age, race/ethnicity, health literacy, and health status [8]. Such factors may, therefore, prevent some traditionally more vulnerable patient populations from realizing the potential benefits of electronic health information transparency [9-11].

The effects of health technology on the digital divide are actively debated. Studies report that nonwhite patients and those with fewer formal years of education are less likely to register for patient portals compared with less vulnerable patient populations [12-19]. However, other studies have found that once patients are enrolled on the patient portal, actual portal use may not differ by race, ethnicity, or socioeconomic status [15]. Although digital disparities exist, some data suggest that the gap is narrowing over time [20,21]. For instance, in 2016, 68% of Internet users had less than a high school education, compared with 41% in 2010 [20]. Between 2010 and 2016, the gap in Internet utilization in general among black users compared with white users nearly closed (85% vs 88%, respectively) [20]. In addition, at least some patients in safety net hospitals are interested in using electronic communication with their providers [22]. Disadvantaged patients may lack access to information needed to make informed care decisions [5], and at the same time, may be at the highest risk of negative health outcomes. Although some experts warn that patient portals and other electronic health (eHealth) innovations may widen disparities in care [12,19], others point out that with literacy-appropriate, user-centered design and better support, such health technology can play an important role to help bridge the divide [4,23].

OpenNotes, a national movement dedicated to making health care more open and transparent by encouraging health care providers to share their visit notes with patients through the patient portal, is giving patients easier access to their medical information [5,24]. As access to visit notes through the patient portal spreads across the country [24,25], we are often asked about whether OpenNotes can benefit vulnerable patients. The question is complicated because the term vulnerable has been broadly interpreted to include any patients who are at risk of health disparities, with respect to race and ethnicity, income, education level, chronic illness, disability, English as a second language, and limited health literacy, among other definitions [26,27]. Little is known about whether and how each of these patient groups may be affected by electronic access to their notes, and whether they view note access as important to their health.

We aimed to better understand how a subset of vulnerable patients engages with their health information. We asked portal-registered patients how important notes are to them for several patient engagement and health behaviors. We hypothesized that patients of different racial and ethnic backgrounds, education levels, and health status may report variable degrees of note importance, and that this information could help guide organizational strategies to engage patients through the patient portal.

## Methods

### Survey Development

We conducted a cross-sectional survey of an adult patient population using the patient portal at an urban US academic medical center. To design the survey, we convened a multidisciplinary team of patients and family members (members of the Patient and Family Advisory Council), health care delivery researchers, nurses, doctors, social workers, and patient engagement and safety experts. This group met regularly to design the survey for 1 year. Survey item structure was adapted from prior published OpenNotes surveys, and new items were developed to focus more specifically on patient engagement and safety [5]. This analysis focused on demographic data and on a subset of survey items assessing patient-perceived importance of notes for various patient engagement activities.

The survey introduction explained open notes and included a screenshot to remind patients where their notes are accessed on the portal. Questions focused on notes (rather than portal use more generally). We asked participants who reported reading at least one note:

How important is reading your notes for:

1. Understanding your health and medical conditions
2. Feeling informed about your care
3. Understanding how your provider(s) are thinking about your medical conditions
4. Remembering the plan for your care (what the provider(s) suggests you do next)
5. Helping you make decisions about your care.

Response options for each item ranged from 0-10, displayed horizontally after each item and anchored with the words not at all important on one end and extremely important at the other. The response scale was selected to allow for greater granularity in assessing the importance of notes to patients of varying demographic backgrounds, as prior data suggested that, overall, the majority of patients supported the idea of OpenNotes [5]. We used administrative data to determine age, sex, number of notes available on the portal, and number of notes accessed.

The survey was reviewed for face validity by members of the Patient and Family Advisory Council and also underwent an external review by a survey scientist with expertise in development of national validated instruments assessing patient and family experience, and it was revised based on their feedback. We then performed formal cognitive testing with 3 additional patients of varying sociodemographic backgrounds. Final survey items relevant to this analysis are shown in Multimedia Appendix 1.

### Participants

Our survey sample consisted of a simple random sample of 31,049 patients at 1 urban US academic health center with active portal accounts and at least one available out-patient visit note available during the prior year. Participants were invited to complete the questionnaire through the patient portal between June 2016 and September 2016 and received up to 2 subsequent reminders. Ten raffle prizes (iPads) were used as incentives for survey participation.

### Analysis

#### Patient Characteristics

We compared demographics, note availability, and note reading between respondents and nonrespondents using the chi-square test for categorical data and the Wilcoxon sign rank test or *t* test for continuous data.

#### Importance of Notes: Bivariate Analysis

We compared the proportion of patients reporting notes were extremely important (designated as 9-10 on a scale of 0-10), by bivariate analysis using the chi-squared test for each of the five health behaviors across the three sociodemographic factors of interest: (1) education, (2) race/ethnicity, and (3) self-reported health.

#### Multivariable Modeling

We used principal component analysis to assess the psychometric properties of the 5 survey items addressing the importance of notes for patient engagement activities. The 5 items showed good internal consistency and represented 1 domain (correlation analysis revealed a Cronbach alpha of .93, see Multimedia Appendix 2). We used a logistic regression model to assess independent demographic and portal use factors associated with reporting that notes are extremely important to engage in care. As our psychometric analysis supported a summary measure, we calculated the mean score of all 5 items, and used a mean summary score of 9-10 as the outcome of interest. We also ran the model for each of the 5 study questions independently to confirm the results. All statistical analyses were performed in SAS version 9.4 (SAS Institute Inc., Cary, NC, USA). The study was approved by our Institutional Review Board.

## Results

### Patient Characteristics

**Figure 1:**
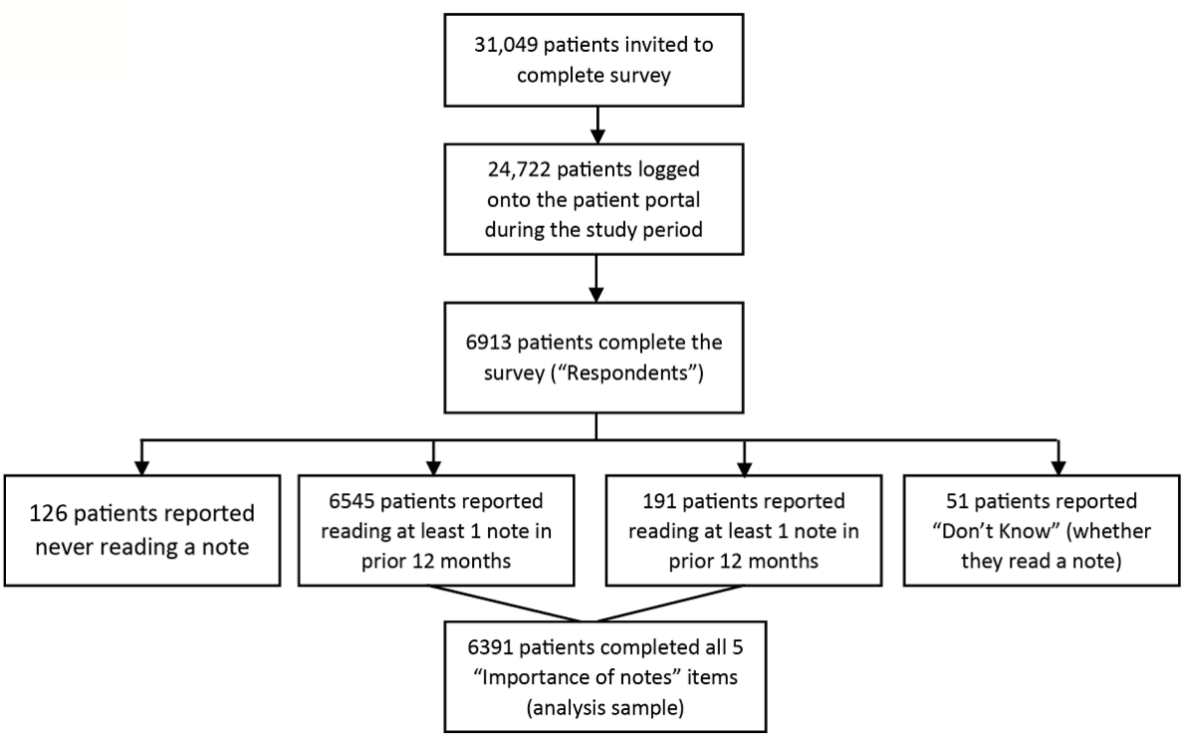
Flowchart of patient recruitment, enrollment, and sampling.

A total of 79.62% (24,722/31,049) invited patients logged on to the portal during the study period and among these, 27.96% (6913/24,722) of patients completed the survey (Figure 1).

The mean age of respondents was 56 years, 62.82% (4343/6913) were women and 82.41% (5697/6913) were white (Table 1). Among participants, 70.40% (4867/6913) reported a bachelor’s degree and 72.21% (4992/6913) held private insurance. Respondents and nonrespondents did not differ by gender and number of hospitalizations. However, compared with nonrespondents, patients who completed the survey were slightly older (51 vs 56 years) and more likely to use Medicare as their primary insurance (17.22%, 4156/24,136 vs 23.65%, 1635/6913). Both groups had a median of 7 notes available, but respondents accessed more notes than nonrespondents (median 4 vs 2). The majority of patients invited to participate in the study were white (77.03%, 23,917/31,049) and college graduates (63.76%, 19,797/31,049), as reflective of the patient population at our academic medical center. Compared with nonrespondents, participants were somewhat more likely to be white (75.49%, 18,220/24,136 vs 82.41%, 5697/6913) and a college graduate (61.86%, 14,930/24,136 vs 70.40%, 4867/6913). Additional participant characteristics and a comparison of respondents versus nonrespondents are shown in Table 1.

### Importance of Notes: Bivariate Analysis

Among all 6913 respondents, 94.68% (6545/6913) reported reading at least one visit note during the prior 12 months, and an additional 2.76% (191/6913) patients read at least one note at some point in the past (126 patients reported never reading a note in the past, and 51 patients reported Don’t Know and were excluded from the importance of note-reading analysis; Figure 1). Among patients who reported reading at least one visit note, 6391 completed all 5 important items and were included in the analysis. The majority of these respondents reported that notes were important for engaging in their care across all five items (Multimedia Appendix 2).

#### Education

Overall, the majority of patients assigned high importance to reading notes, but there were significant differences between patients with varying formal education levels in all 5 survey items (*P*<.001 for all comparisons; Multimedia Appendix 3). Nearly three-fourths of less educated patients reported that notes are extremely important to feel informed about their care (74.0%, 727/982), and to understand how their provider(s) are thinking about their medical conditions (73.3%, 720/982). Compared with respondents with masters or doctorate education, patients with a high school education or less were twice as likely to report that notes are extremely important to remember the plan of care (36.15%, 1069/2957 vs 62.4%, 613/982), and help them make decisions about their care (37.37%, 1105/2957 vs 62.3%, 612/982).

#### Race/Ethnicity

We found significant differences among patients of nonwhite race/ethnicity reporting on the importance of notes for all 5 items (*P* ≤.003 for all comparisons). For example, 64.1% (118/184) of black respondents felt notes were extremely important to understand their health and medical conditions compared with 51.15% (2607/5097) of white respondents. Similarly, 62.0% (114/184) of black patients compared with 41.83% (2132/5097) of white patients reported notes were extremely important to remember the care plan, and 70.7% (130/184) of black patients rated notes as extremely important to understand how the provider is thinking about their medical conditions compared with 59.17% (3016/5097) of white patients. Hispanic/Latino patients and Asian patients also consistently reported higher importance of notes in all categories compared with white patients (Multimedia Appendix 3).

#### Self-Reported Health

Compared with healthier patients, patients with poorer self-reported health were as or more likely to ascribe high importance of notes to understand health conditions, understand how the provider is thinking, remember the care plan, and make decisions about their care (*P* ≤.02 for all comparisons except informed about care; Multimedia Appendix 3). Across all health strata, 62.4% to 65.9% of patients reported notes were extremely important to feel informed about care. Overall, the distribution of responses was U-shaped, with patients reporting excellent health and those reporting fair or poor health being most likely to indicate that notes were extremely important to engage in care, compared with those with very good or good self-reported health. The sickest patients were as, or more, likely to find notes extremely important for engaging in care than the healthiest patients.

### Multivariable Modeling

Each of the variables described in the bivariate analysis remained significant in multivariable modeling (Table 2). Controlling for other demographic characteristics, patients with a high school education or less were more likely than those with more than a college education to report that notes are extremely important to engage in care (odds ratio [OR] 2.9, CI 2.4-3.3, *P*<.001). Similarly, controlling for other characteristics, nonwhite patients were more likely than white patients to report that notes are extremely important to engage in care (OR 2.0, CI 1.5-2.7, *P*<.001 for black patients, and OR 2.2, CI 1.7-2.9, *P*<.001 for Asian patients, and OR 2.2, CI 1.6-2.9, *P*<.001 for Hispanic or Latino patients). As in bivariate analysis, patients with excellent health remained more likely than those with very good or good health to report notes were extremely important. After accounting for race, education, and the other model factors, the sickest patients were as likely to report notes were extremely important as patients with good or very good health, but less likely to do so than those with excellent health (OR 0.7, CI 0.6-0.9, *P*=.001). Females compared with males, older patients compared with younger patients (by each incremental year of age), and patients who accessed more notes compared with those who accessed fewer notes (by each additional note accessed) were also more likely to report that notes were extremely important to engage in care, albeit with smaller effect sizes. Individual models for each of the 5 engagement items and a sensitivity analysis restricting the outcome to a mean of 10 on the summary score (rather than 9-10) revealed similar results (data not shown).

**Table 1:** Comparison of demographics and health care use across safety survey response status.

Demographics^a^ and health care use		Total (n=31,049)	Nonrespondents (n=24,136)	Respondents (n=6913)	*P* value
**Race/ethnicity**					<.001
	Asian, n (%)	1737 (5.59)	1440 (5.97)	297 (4.30)	
	Black, n (%)	1458 (4.70)	1187 (4.92)	271 (3.92)	
	Hispanic/Latino, n (%)	765 (2.46)	653 (2.71)	112 (1.62)	
	White, n (%)	23917 (77.03)	18220 (75.49)	5697 (82.41)	
	Other, n (%)	1531 (4.93)	1217 (5.04)	314 (4.54)	
	Unknown, n (%)	1641 (5.29)	1419 (5.88)	222 (3.21)	
**Age in years**					<.001
	Age, mean (range)	52 (19-101)	51 (19-101)	56 (19-96)	
**Gender**					.40
	Female, n (%)	19371 (62.39)	15028 (62.26)	4343 (62.82)	
	Male, n (%)	11678 (37.61)	9108 (37.74)	2570 (37.18)	
**Education**					<.001
	Less than high school, n (%)	332 (1.07)	300 (1.24)	32 (0.46)	
	High school graduate, n (%)	6566 (21.15)	5233 (21.68)	1333 (19.28)	
	College graduate, n (%)	19797 (63.76)	14930 (61.86)	4867 (70.40)	
	Unknown, n (%)	4354 (14.02)	3673 (15.22)	681 (9.85)	
**Health insurance**					<.001
	Medicaid, n (%)	1663 (5.36)	1390 (5.76)	273 (3.95)	
	Medicare, n (%)	5791 (18.65)	4156 (17.22)	1635 (23.65)	
	Private, n (%)	23,517 (75.74)	18,525 (76.75)	4992 (72.21)	
	Self-pay, n (%)	78 (0.25)	65 (0.27)	13 (0.19)	
**Hospitalization**					.28^b^
	Hospitalizations over prior year (median, range)	0 (0-17)	0 (0-17)	0 (0-12)	
**Notes available**					<.001^b^
	Number of notes available (median, IQR^c^)	7 (4-13)	7 (4-13)	7 (4-14)	
**Notes read**					<.001^b^
	Number of notes read (median, IQR)	3 (1-6)	2 (1-5)	4 (2-8)	

^a^Demographic data taken from administrative sources.

^b^Wilcoxon sign-rank test.

^c^IQR: interquartile range.

**Table 2:** Odds ratios estimated using logistic regression of factors associated with reporting that notes are extremely important for five health behaviors.

Covariates^a^		Odds ratio (95% CI)	*P* value
**Education (reference: masters or doctoral)**			
	Associates or bachelors	1.4 (1.3-1.6)	<.001
	High school graduate or less	2.9 (2.4-3.3)	<.001
**Race/ethnicity (reference: white)**			
	Asian	2.2 (1.7-2.9)	<.001
	Black	2.0 (1.5-2.7)	<.001
	Hispanic or Latino	2.2 (1.6-2.9)	<.001
	Other/multiple races	1.4 (1.1-2.0)	.02
**Self-reported health (reference: excellent)**			
	Fair or poor	0.7 (0.6-0.9)	.001
	Good	0.7 (0.6-0.8)	<.001
	Very good	0.7 (0.6-0.9)	<.001
**Gender (reference: male)**			
	Female	1.3 (1.1-1.4)	<.001
Number of notes read		1.03 (1.02-1.04)	<.001
Age		1.01 (1.01-1.01)	<.001

^a^Observations (n=464) excluded because of missing data.

## Discussion

### Principal Findings and Comparison With Prior Work

Our study of nearly 7000 patient portal users reveals that less educated and nonwhite patients are each independently more likely to report that reading visit notes is extremely important to engage in their care than more educated and white patients. Our study reveals several insights that can help guide future research.

First, although clinicians and health care leaders may expect OpenNotes to most benefit tech-savvy, highly educated patients, our findings suggest that even after controlling for other demographic factors, less educated patients using the portal are nearly three times as likely to report that reading visit notes is extremely important to understand and engage in their care. Nearly three-fourths of patients with a high school education or less rated reading notes as extremely important for being informed about their care and for understanding the doctor’s thought process, and two-thirds reported the same for understanding their health conditions. Our findings resonate with other studies suggesting that although health literacy and access to technology are critical issues [21,28], portal registration may be a key actionable barrier to engagement for at least some patients with fewer years of formal education [14,17]. Among portal users, those with less formal education may find shared notes particularly valuable, perhaps because patients can return to their notes and review information at their own leisure and pace or share them with family or other sources of support after the visit is complete [29,30].

Irrespective of patient’s educational backgrounds, experts estimate that 40%-80% of health visit information is forgotten or misremembered by patients [31]. Information decay is even more pronounced when it is not written or when it is complex. In our study, differences between patients of varying educational backgrounds were particularly stark with respect to the importance of OpenNotes to help patients remember the care plan and make health decisions. These results suggest that sharing notes with patients (and less educated patients in particular) can be important first steps to enhancing adherence and shared decision making; future research focused on these areas is needed.

Second, our findings demonstrate that nonwhite patients were twice as likely to assign extremely high importance to OpenNotes for engaging in their care when compared with white respondents, suggesting that patients of different races and ethnicities may find transparent notes helpful. The health care professionals caring for such patients may also make use of open notes as a way to engage patients of varying backgrounds. Research shows that black patients have more distrust of the health care system compared with white patients, and that this distrust may stem from perceived differences between health care professionals’ values and their own, rather than from their perception of the provider’s competence per se [32,33]. Other ethnic groups such as Asian and Latino populations may also experience distrust [34] or feel that health care providers do not understand their background and values [35]. In our study, over 70% of nonwhite patients reported that reading notes was extremely important to understand how the provider thinks. As greater transparency can lead to greater levels of trust, the invitation to read visit notes may itself strengthen patient-clinician relationships [11]. OpenNotes may provide an opportunity for providers to mitigate distrust by spelling out their thought process. Health care providers who also document a clear understanding of the patient’s concerns and values may take steps toward earning more trust, although further research is needed. However, lack of solicitation or understanding of patient values or use of judgmental language in notes could potentially exacerbate distrust of clinicians and may reinforce patient concerns about divergent priorities. Effects of clinician tone, language, and literacy writing levels in notes requires further study.

Finally, the relationship between self-reported health and importance of notes was more complex. On bivariate analysis, we noted a U-shaped distribution in the data, whereby patients with the highest and lowest self-reported health were most likely to report that notes were extremely important to engage in care across each of the five health behaviors. Overall, roughly two-thirds of patients with a range of poor to excellent health rated shared visit notes as extremely important to feel informed about their care. However, although prior studies suggest that chronically ill patients are more likely to report benefits from personalized health records [21,36], in our study, patients with poorest self-reported health were as likely as those with good or very good health to find notes extremely important for engaging in care, but patients with excellent health were most likely to do so after controlling for race, education, and other demographic factors. This finding may be attributable to several possible factors. For example, our assessment of health did not designate chronically ill patients from others, but rather relied on patient self-reported health, which may not correlate directly with chronic illness. Our population was healthy overall, and we did not have a large enough sample size to distinguish between patients with fair versus poor health, potentially diluting effects by our groupings. In addition, nonwhite patients and those with lower levels of education may have been disproportionately represented among patients with fair or poor health, and race and education demonstrated larger effect sizes. Finally, sicker patients may find notes are not as important because they are too ill or frail to consistently read notes. Instead, informal family and friend caregivers may be the individuals to benefit most from reading notes of sicker patients to remain informed about the patient’s care, as suggested in other studies [37,38]. Future research with larger patient populations who have poor health may help better explore these effects.

About two-thirds (66%) of patients with poor/fair health and 62% of patients with excellent health reported notes were extremely important to understand how the provider is thinking. Better understanding the provider’s thought process may help patients across the health spectrum see the rationale for health recommendations, potentially influencing adherence to treatment plans [39]. As OpenNotes is centered on transparency, and transparency improves trust, it may also be a first step toward improving trust between clinicians and patients, which is itself associated with greater adherence [3,40]. Taken together, our findings reinforce prior studies demonstrating that among patients who use their personalized health record, those with fewer formal years of education and lower income are more likely to feel they have learned about their health care, ask their doctor a question they may not have asked before, or do something specific to improve their health [21,36].

We were intrigued to find that older female patients were more likely to view notes as extremely important, perhaps because women and the elderly may be less assertive, and may find answers to their questions in notes (rather than having to ask the doctor), although the effect was small and this hypothesis requires formal testing. Similarly, patients who read more notes were more likely to report they were extremely important, suggesting that greater use of notes is associated with greater value (perhaps not surprisingly), although the effect size was small. Further research may better differentiate the effects of reading notes for patients who have more visits (and therefore more notes), patients who read notes more frequently, and those who read notes repeatedly.

There is ongoing debate about whether technologic innovations in eHealth will increase or decrease health disparities [3]. Hospitals, health care systems, and clinicians may not prioritize portal registration or other electronic health information engagement for less educated or nonwhite or patients, operating under the assumption that these groups are less likely to benefit. Our findings add to a growing evidence base suggesting that challenging these assumptions may prove important, particularly as health care provider endorsement remains a key predictor of portal use [41,42]. Our findings highlight that at least some patients who are less educated and nonwhite are very interested in accessing their notes on the portal and are two to three times as likely to find them valuable for various patient engagement and health care activities. Better understanding their health conditions may help patients feel better prepared for their visits and more informed for making decisions about their care [5,29]. As trust has been linked with shared decision making in minority populations [43], greater transparency may support patient engagement through stronger relationships with clinicians, provided clinicians use this tool to demonstrate an understanding and appreciation for patients’ values and beliefs.

### Limitations

The study has several important limitations. The survey was conducted at a single US institution, thus limiting generalizability. Although it was a large Internet survey, the majority of respondents were white and educated. However, our data from about 1000 patients with high school education or less, nonwhite race/ethnicity, and or fair or poor self-reported health can help inform future research questions for larger studies of diverse patient populations. The study also had a limited response rate, considering that incentives may drive participation. The responses are likely biased by patients who are more activated (as respondents were already registered on the portal and using OpenNotes)—a limitation that is intrinsic to the study question. Assessing how important notes are to patients for various health engagement behaviors necessitates that patients have read at least one note because responses from patients who never read notes would be hypothetical. As patients had to have an active portal account to receive a study invitation, the perspectives of patients without portal access are not necessarily represented in our data, and more research is needed to better understand and overcome barriers facing vulnerable patient populations who do not register for portals, such as rural patient populations lacking broadband access or those with Internet access who do not enroll on the portal.

Our study focused on patient perceptions of how important OpenNotes are for various health behaviors including understanding health conditions, remembering the care plan, understanding how the provider thinks, feeling informed, and making health decisions, but did not directly assess patient behaviors themselves. Whether access to health information improves adherence and empowers patients to ask questions, voice concerns, or create stronger partnerships with clinicians that enhance shared decision making needs further research. Finally, our study did not assess health literacy. Data show that patients with limited health literacy are less likely to use the portal and will require more active support, outreach, and user-centered approaches [36,44,45], and further research is needed to best engage this population. Moreover, most portals and almost all notes are in English only, although many centers are actively working on translations. Although many patient portals are largely inaccessible for direct use by patients with very limited English proficiency, they may be an important tool for friend or family care givers with whom the patient chooses to share notes (particularly if patients struggle to retain information from the visit or need more time to review and digest it), until reliable translations are routinely available through the portal. Factors affecting vulnerable populations are complex and likely do not operate in isolation. Although we modeled the relative contributions of age, sex race, education, health status, and note access in this first exploration of how some vulnerable patient groups feel about the importance of notes, more nuanced modeling to tease out the contributions of additional factors such as low health literacy, non-English preference, rural versus urban populations, and other cultural or economic influences would be helpful in studies with larger groups of vulnerable patients, including patients in other countries and those with other health insurance systems and access to health care. Such studies may help further to design most effective interventions.

### Conclusions

Sharing health information with at least some vulnerable patient populations through OpenNotes may help engage them. Patients who are less educated and nonwhite are two to three times as likely to assign highest importance to reading their notes for various health behaviors including understanding their health, remembering the care plan, and making informed decisions compared with more educated and white patients. Realizing the possible benefits of OpenNotes for more vulnerable patients may require broad-scale social outreach and portal registration, patient/family and clinician education, and user-friendly portals that are designed in collaboration with diverse end users. Despite these challenges, our study suggests that at least some vulnerable patients are interested in access to their notes and perceive important benefits that may lead to improved engagement and enhanced patient-provider understanding.

## Appendix

Multimedia Appendix 1 Relevant survey items.

Multimedia Appendix 2 Item distribution characteristics and principal component analysis results.

Multimedia Appendix 3 Percent of patients reporting notes are extremely important for each of five health behaviors by patient demographics.

## Abbreviations

eHealth: electronic health
EHR: electronic health record
IQR: interquartile range
OR: odds ratio
