# Supplementary material for: The Importance of Visit Notes on Patient Portals for Engaging Less Educated or Nonwhite Patients: Survey Study
Source: J Med Internet Res. 2018 May 24;20(5):e191. doi: 10.2196/jmir.9196 (PMC5992450; doi:10.2196/jmir.9196)
Supplement: Multimedia Appendix 1 [file jmir_v20i5e191_app1.pdf]

Appendix 1: Relevant Survey Items

How important is reading your notes in understanding your health and medical conditions?

Not at all important										Extremely important
0	1	2	3	4	5	6	7	8	9	10
☐	☐	☐	☐	☐	☐	☐	☐	☐	☐	☐

How important is reading your notes in feeling informed about your care?

Not at all important										Extremely important
0	1	2	3	4	5	6	7	8	9	10
☐	☐	☐	☐	☐	☐	☐	☐	☐	☐	☐

How important is reading your notes in understanding how your provider(s) are thinking about your medical conditions?

Not at all important										Extremely important
0	1	2	3	4	5	6	7	8	9	10
☐	☐	☐	☐	☐	☐	☐	☐	☐	☐	☐

How important is reading your notes in remembering the plan for your care (what your provider(s) suggests you do next)?

Not at all important										Extremely important
0	1	2	3	4	5	6	7	8	9	10
☐	☐	☐	☐	☐	☐	☐	☐	☐	☐	☐

How important is reading your notes in helping you make decisions about your care?

Not at all important										Extremely important
0	1	2	3	4	5	6	7	8	9	10
☐	☐	☐	☐	☐	☐	☐	☐	☐	☐	☐
