# Supplementary material for: The Importance of Visit Notes on Patient Portals for Engaging Less Educated or Nonwhite Patients: Survey Study
Source: J Med Internet Res. 2018 May 24;20(5):e191. doi: 10.2196/jmir.9196 (PMC5992450; doi:10.2196/jmir.9196)
Supplement: Multimedia Appendix 2 [file jmir_v20i5e191_app2.pdf]

Appendix 2. Item distribution characteristics and principal component analysis results.

Item	Mean	Standard deviation	Factor loading
How important is reading your notes in:			
Understanding your health and medical conditions?	8.1	2.3	0.914
Feeling informed about your care?	8.5	2.2	0.902
Understanding how your provider(s) are thinking about your medical conditions?	8.4	2.2	0.881
Remembering the plan for your care (what your provider(s) suggests you do next)?	7.2	3.0	0.834
Helping you make decisions about your care?	7.3	2.9	0.890

NOTE: 1 factor was kept for PCA based on a minimum Eigenvalue criteria of 1. Chronbach alpha for the 5 items is 0.934.
