# Supplementary material for: The Importance of Visit Notes on Patient Portals for Engaging Less Educated or Nonwhite Patients: Survey Study
Source: J Med Internet Res. 2018 May 24;20(5):e191. doi: 10.2196/jmir.9196 (PMC5992450; doi:10.2196/jmir.9196)
Supplement: Multimedia Appendix 3 [file jmir_v20i5e191_app3.pdf]

Multimedia Appendix 3. Percent of patients reporting notes are extremely important for each of 5 health behaviors by patient demographics.

**Demographic***	**Understanding health conditions**		**Informed about care**		**Understanding how provider thinks**		**Remembering care plan**		**Making care decisions**		**Mean summary score 9-10 (extremely important) across all 5 behaviors**	
**Characteristic**	**n (%)**	***P value***	**n (%)**	***P value***	**n (%)**	***P value***	**n (%)**	***P value***	**n (%)**	***P value***	**n (%)**	***P value***
**Education**												
≤High School N=982	649(66.1)	<.0001	727(74.0)	<.0001	720(73.3)	<.0001	613(62.4)	<.0001	612(62.3)	<.0001	601(61.2)	<.0001
Associates or Bachelors N=2115	1104(52.20)		1332(62.98)		1266(59.86)		989(46.76)		959(45.34)		915(43.26)	
Masters or Doctoral N=2957	1423(48.12)		1783(60.30)		1666(56.34)		1069(36.15)		1105(37.37)		1047(35.41)	
**Race/Ethnicity**												
Asian N=256	164(64.1)	<.0001	181(70.7)	.0028	166(64.8)	.0001	142(55.5)	<.0001	144(56.3)	<.0001	138(53.9)	<.0001
Black N=184	118(64.1)		130(70.7)		130(70.7)		114(62.0)		110(59.8)		109(59.2)	
Hispanic or Latino N=219	130(59.4)		153(69.9)		151(69.0)		135(61.6)		128(58.5)		126(57.5)	
White N=5097	2607(51.15)		3188(62.55)		3016(59.17)		2132(41.83)		2154(42.26)		2053(40.28)	
Other/Multiple Races N=186	106(57.0)		123(66.1)		124(66.7)		96(51.6)		90(48.4)		92(49.5)	
**Self-reported Health**												
Excellent N=918	503(54.8)	.0026	604(65.8)	.1074	569(62.0)	.0007	410(44.7)	.0076	412(44.9)	.0180	413(45.0)	.0023
Very good N=2439	1237(50.72)		1524(62.48)		1411(57.85)		1025(42.03)		1025(42.03)		974(39.93)	
Good N=1962	1009(51.43)		1224(62.39)		1183(60.30)		874(44.55)		884(45.06)		831(42.35)	
Fair/Poor N=757	437(57.7)		499(65.9)		499(65.9)		371(49.0)		364(48.1)		354(46.8)	

*NOTE: All P-values from chi-square test. Of the 6391 patents included in the analysis 337 were missing data on education, 315 were missing data on general health and 449 were missing data on race/ethnicity.
